# Microfluidic Devices for Biomedical Applications: Biomedical Microfluidic Devices 2019

**DOI:** 10.3390/mi11040370

**Published:** 2020-04-01

**Authors:** Kwang W. Oh

**Affiliations:** SMALL (Sensors and MicroActuators Learning Lab), Department of Electrical Engineering & Department of Biomedical Engineering, University at Buffalo, State University of New York (SUNY-Buffalo), Buffalo, NY 14260, USA; kwangoh@buffalo.edu; Tel.: +1-716-645-1025

Microfluidic devices and systems are well-suited for the manipulation of biomolecules, cells, or particles. These microfluidic devices are capable of a variety of functions to replace routine biomedical analysis and diagnostics, highlighting a higher-level system integration with improved potential for automation, control, and high-throughput processing, while consuming a small volume of samples and reagents at shorter bioassay times and reduced cost [1]. This Special Issue of *Micromachines*, entitled Biomedical Microfluidic Devices 2019, provides a discussion of the technical challenges associated with developing microfluidic devices for biomedical applications. Addressing these challenges requires technological advances in many areas, including sensing [2,3,4]; cell manipulation [5,6]; cell, tissue, and organ culture platforms [7,8,9]; and micropumping technologies [10]. This Special Issue consists of nine high-quality papers, including one insightful review article [2]. 

*Sensors*: On-chip sensors integrated with microfluidic devices have great potential in lab-on-chip or stand-alone systems for various biological and biomedical applications. A review article by Fu et al. [2] summarizes the fundamental configuration of a device combining solid-state nanopores and microfluidic systems, detection improvement from a multichannel approach, multifunctional detection resulting from an optical–electrical detection method, high-level integration, and a prototype for commercialization. Using impedance spectroscopy, Karimi et al. [3] report the study of the impedance behavior of platelet-poor plasma (PPP) and platelet-rich plasma (PRP) in a microflow chamber with interchangeable biomimetic surfaces to evaluate global hemostasis. Lin et al. [4] study a simple microfluidic device using planar electrodes for the measurement of the imaginary part of the Clausius–Mossotti factor, Ki, for cells and particles involved in electrorotation (ER) and travelling wave dielectrophoresis (twDEP). The measurement of Ki would be beneficial for the study of the torque in ER, the force in twDEP, the dielectric properties of cells, or physical phenotyping of different cells. 

*Cell manipulation*: The manipulation of individual cells of interest is critical to further studies of phenotypic heterogeneity occurring due to molecular and cellular stochastic processes, phases of cells, asymmetric partitioning during cell division, and inhomogeneous cellular environments. To overcome this challenge, Kumar et al. [5] present a digital microfluidic droplet device for single bacterium capture and selective retrieval from a population using optical tweezers. Among cell manipulation techniques, the use of inertial microfluidics using no external forces is of great interest, because microfluidic systems are simple and suitable for high-throughput cell sorting and analysis. Suwannaphan et al. [6] studied two inertial separation techniques using a spiral microchannel and a contraction–expansion array for the investigation of leukocyte viability and damage, along with the separation efficiency of the devices.

*Cell, tissue, and organ culture platforms*: Cultured cells in Petri dishes and tissue culture flasks (i.e., in vitro) experience completely different environmental cues compared to natural tissues within a complex three-dimensional extracellular matrix (i.e., in vivo), resulting in radical variations in cell morphology and function. Miniaturized culture systems (i.e., ex vivo) are capable of more advanced tissue functions as compared to any current in vivo studies. This allows for more accurate study of complex mechanisms underlying tissue growth, renewal, and disease than any current in vitro models, without the difficulties inherent to in vivo studies. Baydoun et al. [7] present ex vivo intestinal tissue culture models to cultivate full-thickness murine colon explants, which maintain the morphological structures of the tissues for up to eight days. Additionally, they studied numerical simulation of the transport of oxygen and glucose to improve the functionality of biomedical microfluidic devices. Luo et al. [8] designed an integrated microfluidic device for culture of individual coral polyps, featuring a uniform flow environment, rapid mass transfer, and precise temperature control. The microfluidic device provided a reliable analytical approach for model and mechanism investigations of coral bleaching and reef conservation. Williams et al. [9] tackled one of the troublesome issues in microfluidic cell culture platforms, whereby undesired trapping of air bubbles in the passage often causes cell damage or device delamination. To prevent air bubble entry into microfluidic channels, they demonstrated a debubbler module that can rapidly remove bubble volumes spanning three orders of magnitude from segmented flows, at flow rates compatible with those required for microfluidic shear stimulation studies. 

*Micropumping*: One feature that is highly demanded in biomedical lab-on-chip or point-of-care devices is compact, robust, self-driven micropumping without any complex external systems. To overcome the challenges associated with this feature, a variety of pumping methods have been developed, such as finger-actuated pumping, capillary pumping, gravity-driven pumping, and pre-degassed pumping. Among these, a vacuum-assisted pumping method utilizing the gas permeability or solubility of polydimethylsiloxane (PDMS) is extremely suitable for many biomedical microfluidic systems due to its simple implementation. Wang et al. [10] introduced a compact, syringe-assisted, vacuum-driven micropumping module that can be easily attached to the outlet of any existing microfluidics device, providing greater flexibility in many biomedical applications.

I am sure that this Special Issue will be of high interest for many areas of research involved in the multidisciplinary field of biomedical microfluidic devices. I look forward to you sharing your stories of technical challenges and great solutions in the additional Special Issue of *Micromachines*: Biomedical Microfluidic Devices 2020.
